# Integrative network toxicology analysis reveals mechanistic links between PFOA exposure and male reproductive dysfunction

**DOI:** 10.1186/s12610-026-00328-y

**Published:** 2026-09-28

**Authors:** Tao Zhou, Xinyu Zhang, Jinghong Yang, Juan Wang

**Affiliations:** 1 Department of Urology, Santai Hospital Affiliated to North Sichuan Medical College, Mianyang, Sichuan 621100 China; 2 Department of Urology, Affiliated Hospital of Southwest Medical University, Luzhou, Sichuan 646000 China; 3 Department of Orthopedics, Affiliated Hospital of Southwest Medical University, Luzhou, Sichuan 646000 China; 4 Department of Breast and Thyroid Surgery, The First Affiliated Hospital of Chongqing Medical University, Chongqing, 404100 China

**Keywords:** Perfluorooctanoic acid, Erectile dysfunction, Male infertility, Testicular dysfunction, Reproductive toxicology, Acide perfluorooctanoïque, Dysfonction érectile, Infertilité masculine, Dysfonction testiculaire, Toxicologie de la reproduction

## Abstract

**Objective:**

To systematically investigate the molecular mechanisms linking perfluorooctanoic acid (PFOA) to male reproductive health outcomes within a network toxicology framework, and to compare shared pathways across erectile dysfunction (ED), male infertility, and testicular dysfunction.

**Methods:**

PFOA associated targets were identified using CTD, SwissTargetPrediction, and TargetNet, while disease related genes were collected from GeneCards. Protein–protein interaction (PPI) networks were constructed using STRING and analyzed in Cytoscape to identify hub genes. GO and KEGG enrichment analyses were performed to characterize biological processes and pathways affected by PFOA. Molecular docking was used to assess binding affinities between PFOA and key target proteins. In addition, preliminary in vitro experiments were performed to examine the expression patterns of selected candidate genes following PFOA exposure.

**Results:**

A total of 105 PFOA related genes were identified. Intersection analyses yielded 15, 25, and 42 genes associated with ED, male infertility, and testicular dysfunction, respectively. Ten core genes (AR, ESR1, BCL2, NOS1, NOS2, NOS3, ACE, SIRT1, TLR9, SHBG) were shared across all phenotypes and showed high connectivity in the PPI network. Enrichment analyses indicated involvement in reproductive system development, hormone and nuclear receptor regulation, calcium and nitric oxide (NO) signaling, mitochondrial and redox processes, and apoptosis and inflammation related pathways. Preliminary in vitro experiments further validated the expression changes of selected key genes.

**Conclusions:**

This study identifies key molecular targets and pathways potentially mediating PFOA associated male reproductive toxicity. The results support a working hypothesis that PFOA associated male reproductive toxicity may involve altered androgen estrogen receptor signaling, NO related pathways, and apoptosis/inflammation associated regulation. These findings provide a theoretical basis for future mechanistic validation and risk assessment.

## Résumé

**Objectif:**

Étudier de manière systématique, dans le cadre de la toxicologie des réseaux, les mécanismes moléculaires reliant l’acide perfluorooctanoïque (PFOA) aux effets sur la santé reproductive masculine, et comparer les voies communes associées à la dysfonction érectile (DE), à l’infertilité masculine et au dysfonctionnement testiculaire.

**Méthodes:**

Les cibles associées au PFOA ont été identifiées à l’aide de CTD, SwissTargetPrediction et TargetNet, tandis que les gènes liés à la maladie ont été recueillis dans GeneCards. Des réseaux d’interactions protéine-protéine (IPP) ont été construits à l’aide de STRING et analysés dans Cytoscape afin d’identifier les gènes pivots. Des analyses d’enrichissement GO et KEGG ont été réalisées pour caractériser les processus biologiques et les voies affectés par le PFOA. L’amarrage moléculaire a été utilisé pour évaluer les affinités de liaison entre le PFOA et les principales protéines cibles. En outre, des expériences préliminaires in vitro ont été menées afin d’étudier les profils d’expression de certains gènes candidats après exposition au PFOA.

**Résultats:**

Au total, 105 gènes liés à la PFOA ont été identifiés. Les analyses d’intersection ont mis en évidence respectivement 15, 25 et 42 gènes associés à la dysfonction érectile, à l’infertilité masculine et à la dysfonction testiculaire. Dix gènes centraux (AR, ESR1, BCL2, NOS1, NOS2, NOS3, ACE, SIRT1, TLR9, SHBG), communs à l’ensemble des phénotypes, présentaient une forte connectivité dans le réseau d’interactions protéine-protéine. Les analyses d’enrichissement ont révélé leur implication dans le développement de l’appareil reproducteur, la régulation hormonale et celle des récepteurs nucléaires, la signalisation calcique et celle du monoxyde d’azote (NO), les processus mitochondriaux et d’oxydoréduction, ainsi que les voies liées à l’apoptose et à l’inflammation. Des expériences préliminaires in vitro ont en outre confirmé les variations d’expression de certains gènes clés.

**Conclusions:**

Cette étude identifie des cibles moléculaires et des voies de signalisation clés susceptibles de contribuer à la toxicité du PFOA sur la fonction reproductive masculine. Les résultats étayent l’hypothèse de travail selon laquelle cette toxicité pourrait impliquer une altération de la signalisation des récepteurs aux androgènes et aux œstrogènes, des voies liées au NO ainsi que des mécanismes de régulation associés à l’apoptose et à l’inflammation. Ces résultats fournissent une base théorique pour de futures validations mécanistiques et évaluations des risques.

## Introduction

Male reproductive health has become a major global public health concern [1]. The prevalence of male infertility, ED, and testicular dysfunction has increased over recent years, imposing substantial burdens on individuals, families, and society [2]. Multiple environmental factors are considered important contributors to male reproductive impairment, including chemical pollutants, lifestyle factors, dietary habits, and occupational exposures [3]. Among these, chemical pollutants have attracted intense attention due to their ubiquity, persistence, resistance to degradation, and propensity for bioaccumulation [4, 5]. Of particular concern are endocrine-disrupting chemicals (EDCs), which can mimic or block hormonal signaling, interfere with hormone synthesis and metabolism, and ultimately affect spermatogenesis, sexual function, and testicular structure and function [6–8]. Accumulating evidence indicates that long-term exposure to EDCs may lead to reduced sperm quantity and quality, testicular atrophy, abnormal hormone levels, and sexual dysfunction, highlighting the potential contribution of environmental chemicals to male reproductive disorders [9, 10].

Perfluorooctanoic acid (PFOA), a typical member of per- and polyfluoroalkyl substances, has been widely used since the mid-twentieth century in water-, oil-, and stain-resistant coatings, textiles, anti-fouling agents, and various consumer products [11, 12]. Owing to its exceptional chemical stability, environmental persistence, and bioaccumulative potential in water, soil, and biota, PFOA has been referred to as a “forever chemical” [13]. Numerous studies have detected PFOA in human and environmental samples, indicating widespread exposure risk [14]. Syntheses of animal and epidemiological studies suggest that PFOA may exert toxic effects on the male reproductive system [15]. A meta-analysis summarizing 16 animal studies reported associations between PFOA exposure and decreased serum testosterone, abnormal sperm parameters, altered absolute/relative weights of the testis and epididymis, and histopathological changes in testicular tissue [16]. These findings indicate that PFOA associated reproductive toxicity is not incidental but shows a degree of consistency and reproducibility.

Nevertheless, despite accumulating evidence supporting the adverse effects of PFOA on male reproductive health, important knowledge gaps remain [17]. First, most previous studies have primarily described associations between PFOA exposure and reproductive outcomes, whereas the underlying molecular mechanisms remain insufficiently understood [16]. Second, existing research has largely focused on individual reproductive endpoints and has rarely evaluated multiple male reproductive phenotypes within a unified framework [18]. Therefore, systematic integration of different forms of PFOA associated reproductive impairment is still lacking, which limits a comprehensive assessment of overall male reproductive risk.

Network toxicology integrates toxicants, targets, and disease pathways in a systems-level manner, providing a powerful strategy to explore complex toxicological mechanisms. Compared with traditional toxicology approaches that rely on single targets or single pathways and are often time-consuming and costly, network toxicology integrates information from toxicological databases, predicted chemical targets, protein interaction networks, and functional annotation resources to characterize chemical–target–pathway–disease relationships at a systems level. In this study, we applied a network toxicology approach to investigate the relationships between PFOA and three male reproductive health phenotypes, aiming to elucidate shared mechanisms and provide a more comprehensive theoretical basis for risk assessment, mechanistic research, and public health interventions.

## Materials and methods

### Toxicity prediction of PFOA

The simplified molecular-input line-entry system (SMILES) string of PFOA was retrieved from PubChem by searching “Perfluorooctanoic Acid.” The SMILES string was then entered into the admetSAR 3.0 online platform to predict its reproductive toxicity potential [19].

### Identification of PFOA- and disease related targets

Putative targets of PFOA were retrieved and predicted using CTD, SwissTargetPrediction, and TargetNet. Specifically, PFOA was searched in CTD using “Perfluorooctanoic Acid,” and genes associated with “Erectile Dysfunction,” “Infertility, Male,” and “Testicular Diseases” were extracted under the “Diseases” section [20]. In SwissTargetPrediction, the SMILES string of PFOA was submitted, and the species was set to *Homo sapiens* for target prediction. In TargetNet, parameters were set as previously described [21], with an area under the curve (AUC) ≥0.7 and restriction to human genes to obtain candidate PFOA associated targets. Targets obtained from the three resources were merged and deduplicated to generate the final PFOA related gene set for downstream network construction and functional analyses.

Disease associated gene lists were collected from GeneCards by searching “Erectile Dysfunction,” “Male infertility,” and “Testicular dysfunction.” Based on the relevance score, the top 50% of genes were retained as disease associated genes for each phenotype [22]. The intersection between the PFOA related gene set and each disease associated gene set was then computed to obtain PFOA–disease–related genes.

### Construction of the PPI Network

A PPI network was constructed using the PFOA–disease related genes [23]. Gene lists were input into the STRING database (species set to *Homo sapiens*), with the interaction confidence score threshold set to ≥0.4. Isolated nodes without interactions were excluded to improve network reliability and interpretability. The resulting network was imported into Cytoscape (version 3.10) for visualization and analysis. The built-in NetworkAnalyzer plugin was used for topological analysis, including calculation of degree, clustering coefficient, and shortest path. Degree represents the number of connections between a node (gene/protein) and other nodes, reflecting its centrality and influence in the network. Hub genes were identified by ranking nodes according to degree; highly connected hub genes were considered more likely to play central regulatory roles in PFOA associated reproductive toxicity [24].

### Enrichment analysis

Functional annotation and pathway enrichment analyses were performed for the PFOA–disease–related genes. GO enrichment analysis was conducted to characterize biological processes, molecular functions, and cellular components [25]. KEGG pathway enrichment analysis was conducted to identify implicated signaling and metabolic pathways [26]. Statistical significance for enrichment was evaluated using hypergeometric-based tests (e.g., Fisher’s exact test). To control the false positive rate due to multiple testing, raw *p* values were adjusted using the Benjamini–Hochberg false discovery rate (FDR) procedure. Significant GO terms and KEGG pathways were ranked by adjusted *p* values, and the top 10 entries were visualized using bar plots or bubble plots to facilitate biological interpretation.

### Molecular docking

The three-dimensional (3D) structure of PFOA was downloaded from PubChem. Protein structures of key target genes were downloaded from the Research Collaboratory for Structural Bioinformatics Protein Data Bank (RCSB PDB). Molecular docking was performed using CB-Dock2. A Vina score < 0 indicates favorable binding; a Vina score < −5 suggests relatively stable binding; and a Vina score < −7 suggests tight binding [27].

### Cell culture

Leydig cells (Procell, China) and rat corpus cavernosum endothelial cells (CCECs) (Procell, China) were used in the present study. Both cell types were cultured in appropriate complete medium supplemented with fetal bovine serum and penicillin/streptomycin at 37 °C in a humidified incubator containing 5% CO₂. Cells in good growth condition and at approximately 80% confluence were used for subsequent experiments. Before treatment, cells were seeded into 6-well plates at a density of 2 × 10^5 cells/well. According to the experimental design, the cells were randomly assigned to four groups: the normal control group (NC), cultured in regular medium; the 5 μM PFOA group, treated with 5 μM perfluorooctanoic acid (PFOA; Aladdin, China); the 10 μM PFOA group, treated with 10 μM PFOA; and the 20 μM PFOA group, treated with 20 μM PFOA.

### RT-qPCR assay

Total RNA was extracted from Leydig cells and rat corpus cavernosum endothelial cells using a total RNA extraction kit according to the manufacturer’s instructions, and RNA concentration was determined using a NanoDrop spectrophotometer (Thermo Scientific, USA). Reverse transcription was performed using 1 μg of total RNA with a commercial reverse transcription master mix according to the manufacturer’s protocol.

Quantitative PCR was carried out using gene-specific primers and SYBR Green master mix in a 10 μL reaction volume. Amplification was performed on an ABI 7500 real-time PCR system (Thermo Scientific, USA) under the following cycling conditions: 95 °C for 60 s, followed by 35 cycles of 95 °C for 15 s, 60 °C for 30 s, and 72 °C for 30 s. Relative gene expression was calculated using the comparative Ct (2^-ΔΔCt) method.

### Statistical analysis

Data are expressed as the mean ± standard deviation (SD) based on at least three independent experiments. Statistical analyses were conducted using GraphPad Prism 10 (GraphPad Software, San Diego, CA, USA) and R software (version 4.2.0).

Differences among multiple groups were assessed by one-way analysis of variance (ANOVA) with Tukey’s multiple-comparison test. When the assumptions of normality or homogeneity of variance were not satisfied, the Kruskal–Wallis test was employed as a nonparametric alternative.

## Results

### Toxicity prediction of PFOA

**Fig. 1:**
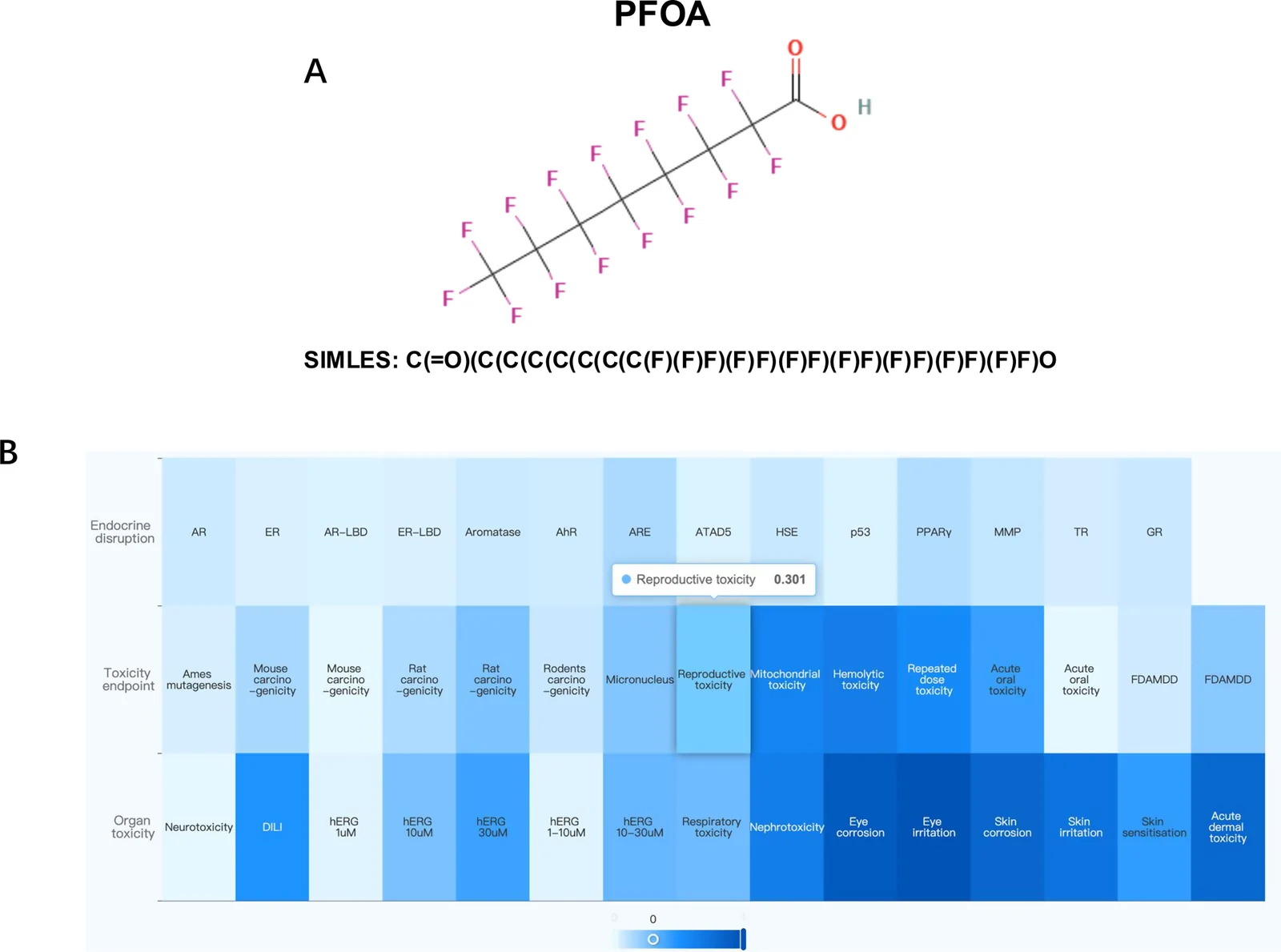
Structural characteristics and predicted toxicological profile of PFOA. **A.** Chemical structure and SMILES notation of PFOA. **B.** Predicted reproductive toxicity potential of PFOA using the admetSAR 3.0 online platform

The chemical structure of PFOA was obtained from PubChem (Fig. 1A). Toxicity prediction suggested a reproductive toxicity probability of 30.1%, indicating that PFOA may possess potential reproductive toxicity (Fig. 1B).

### Potential effects of PFOA on ED

**Fig. 2:**
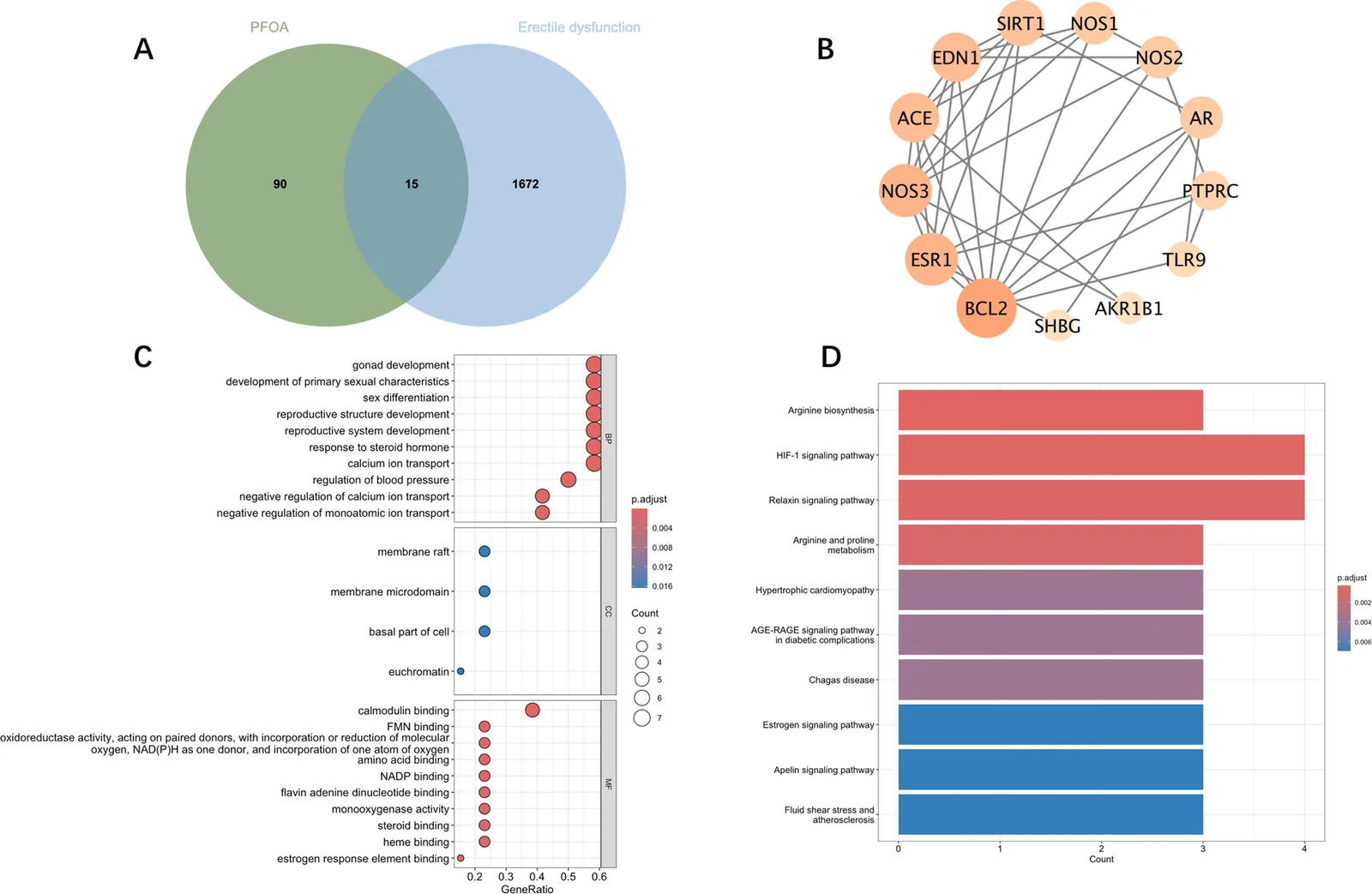
Identification and functional analysis of PFOA associated genes related to erectile dysfunction. **A.** The venn diagram illustrates the intersection of target genes associated with PFOA and ED related genes. **B.** PPI network of the hub genes in PFOA-ED related genes. **C.** GO enrichment analysis of PFOA-ED related genes. **D.** KEGG pathway enrichment analysis of PFOA-ED related genes

We obtained 105 PFOA related genes and 1,687 ED related genes; intersection analysis identified 15 PFOA–ED related genes (Fig. 2A). The PPI network suggested that BCL2, hormone receptor related nodes, and the nitric oxide synthase family may play important roles (Fig. 2B).

GO enrichment results (Fig. 2C) showed significant enrichment of PFOA–ED related genes in multiple biological processes closely associated with reproductive function and erectile physiology, including gonad development, sex differentiation, reproductive structure formation, and reproductive system development. These genes were also enriched in responses to steroid hormones, regulation of calcium ion transport (including negative regulation), and blood pressure regulation—processes related to penile hemodynamics. In terms of cellular components, these genes were mainly associated with membrane rafts, membrane microdomains, and the basal part of cells, suggesting potential involvement in receptor-mediated signaling, endothelial function, or smooth muscle status. For molecular functions, enrichment included calmodulin binding, binding to flavin mononucleotide/flavin adenine dinucleotide/nicotinamide adenine dinucleotide (phosphate), oxidoreductase activity, and monooxygenase activity, as well as steroid binding and estrogen response element binding. Overall, PFOA may influence key molecules and pathways relevant to ED by disrupting hormonal signaling, calcium homeostasis, redox balance, and critical reproductive developmental processes.

KEGG enrichment analysis (Fig. 2D) indicated that PFOA–ED related genes were enriched in multiple pathways relevant to reproductive function and erectile physiology. Arginine biosynthesis and arginine/proline metabolism were significantly enriched, suggesting that PFOA may affect NO synthesis and endothelium-dependent vasodilation, a central mechanism in ED. In addition, the hypoxia-inducible factor 1 signaling pathway and the relaxin signaling pathway were enriched; both are involved in endothelial homeostasis, hypoxia responses, and vascular smooth muscle regulation, supporting the possibility that PFOA may affect erectile function by disrupting hemodynamic regulation. Other enriched pathways included estrogen signaling, apelin signaling, and fluid shear stress and atherosclerosis, which are related to hormonal regulation, vascular reactivity, and endothelial injury. Enrichment of the advanced glycation end-product–receptor for advanced glycation end-product signaling pathway and cardiac hypertrophy related pathways further suggested that PFOA exposure may exacerbate ED related dysfunction through a metabolism–cardiovascular–endothelial injury axis.

### Potential effects of PFOA on male infertility

**Fig. 3:**
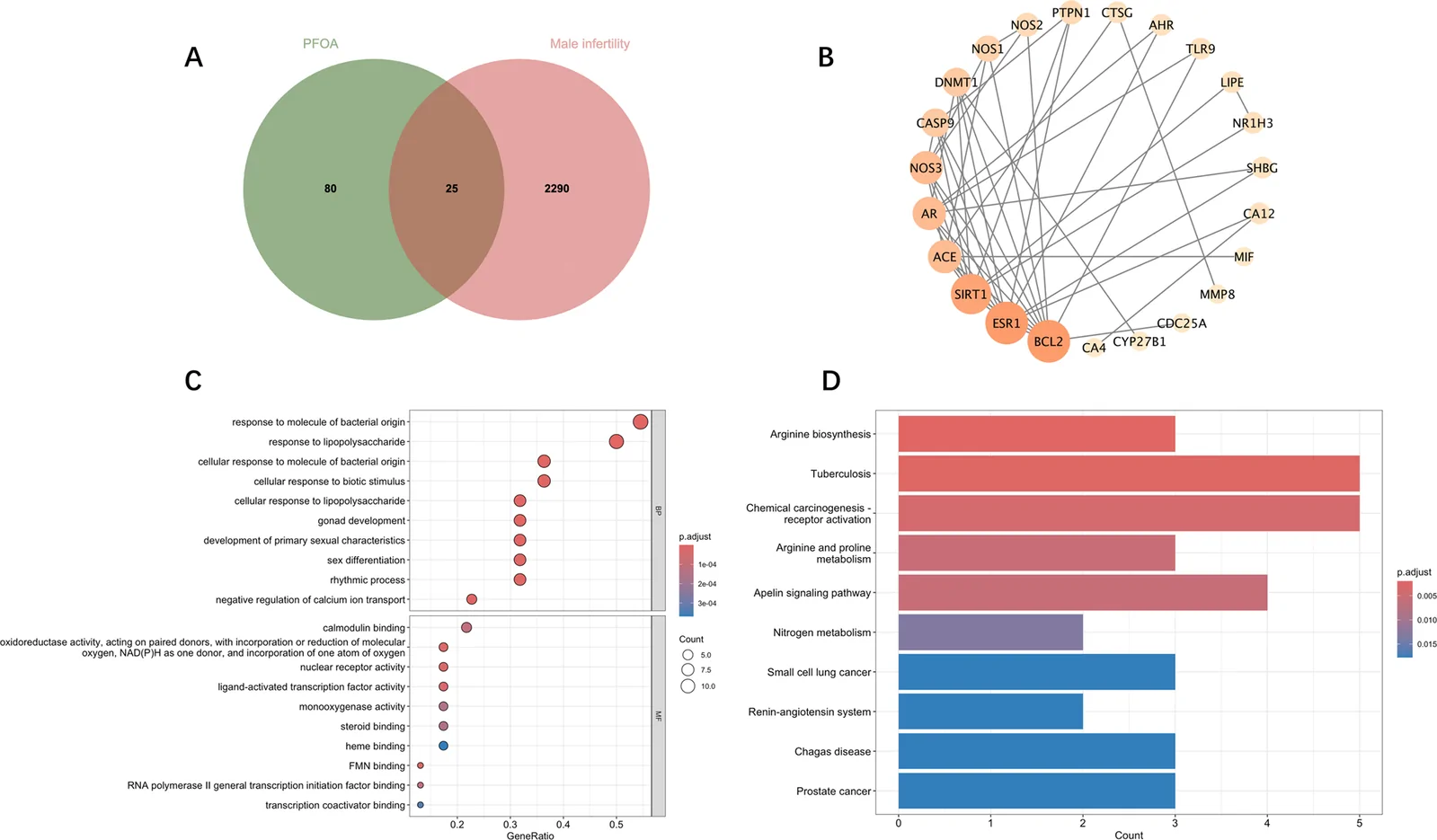
Identification and functional analysis of PFOA associated genes related to male infertility. **A.** The venn diagram illustrates the intersection of target genes associated with PFOA and male infertility related genes. **B.** PPI network of the hub genes in PFOA- male infertility related genes. **C.** GO enrichment analysis of PFOA- male infertility related genes. **D.** KEGG pathway enrichment analysis of PFOA- male infertility related genes

We obtained 105 PFOA related genes and 2,315 male infertility related genes; intersection analysis identified 25 PFOA–male infertility–related genes (Fig. 3A). The PPI network suggested that apoptosis associated molecules, hormone receptor related nodes, and nitric oxide synthase family members were central components (Fig. 3B).

GO enrichment analysis (Fig. 3C) showed significant enrichment in biological processes and molecular functions. In biological processes, genes were enriched in responses to bacterial-derived molecules and lipopolysaccharide (LPS), and cellular responses to biological stimuli—indicating that PFOA may affect male fertility by inducing inflammatory responses or immune dysregulation in the reproductive system. Enrichment was also observed in gonadal development, primary sexual characteristic development, and sex differentiation, suggesting interference with key processes in spermatogenesis and male reproductive development. Notably, enrichment in rhythmic processes and negative regulation of calcium ion transport suggested that PFOA may further aggravate reproductive impairment through disruption of endocrine rhythms and calcium signaling homeostasis. In molecular functions, candidate genes were enriched in calmodulin binding, oxidoreductase activity, nuclear receptor activity, ligand-activated transcription factor activity, monooxygenase activity, steroid binding, heme binding, and flavin mononucleotide binding, supporting potential perturbation of steroid hormone synthesis, cytochrome P450 function, and energy metabolism. Enrichment in RNA polymerase II promoter binding and transcription coactivator binding also suggested that PFOA may influence key transcriptional regulatory processes and alter the expression patterns of reproduction related genes.

KEGG enrichment analysis (Fig. 3D) indicated that key genes were enriched in multiple metabolic and signaling pathways related to male reproductive function. Arginine biosynthesis, arginine/proline metabolism, and nitrogen metabolism were significantly enriched, suggesting that PFOA may disrupt NO synthesis and metabolic homeostasis; NO is important for maintaining the testicular microenvironment, spermatogenesis, and male fertility. Enrichment of the renin–angiotensin system and the apelin signaling pathway suggested that PFOA may affect testicular blood flow regulation, oxidative stress, and endothelial function, thereby indirectly impairing reproductive function. Several disease related pathways were also enriched, including prostate cancer, small cell lung cancer, chemical carcinogenesis, tuberculosis, and Chagas disease. These pathways are often associated with chronic inflammation, immune dysregulation, DNA damage responses, and apoptosis imbalance, implying that PFOA may increase vulnerability of the male reproductive system by inducing inflammation, promoting genetic damage, and disturbing cellular homeostasis.

### Potential effects of PFOA on testicular dysfunction

**Fig. 4:**
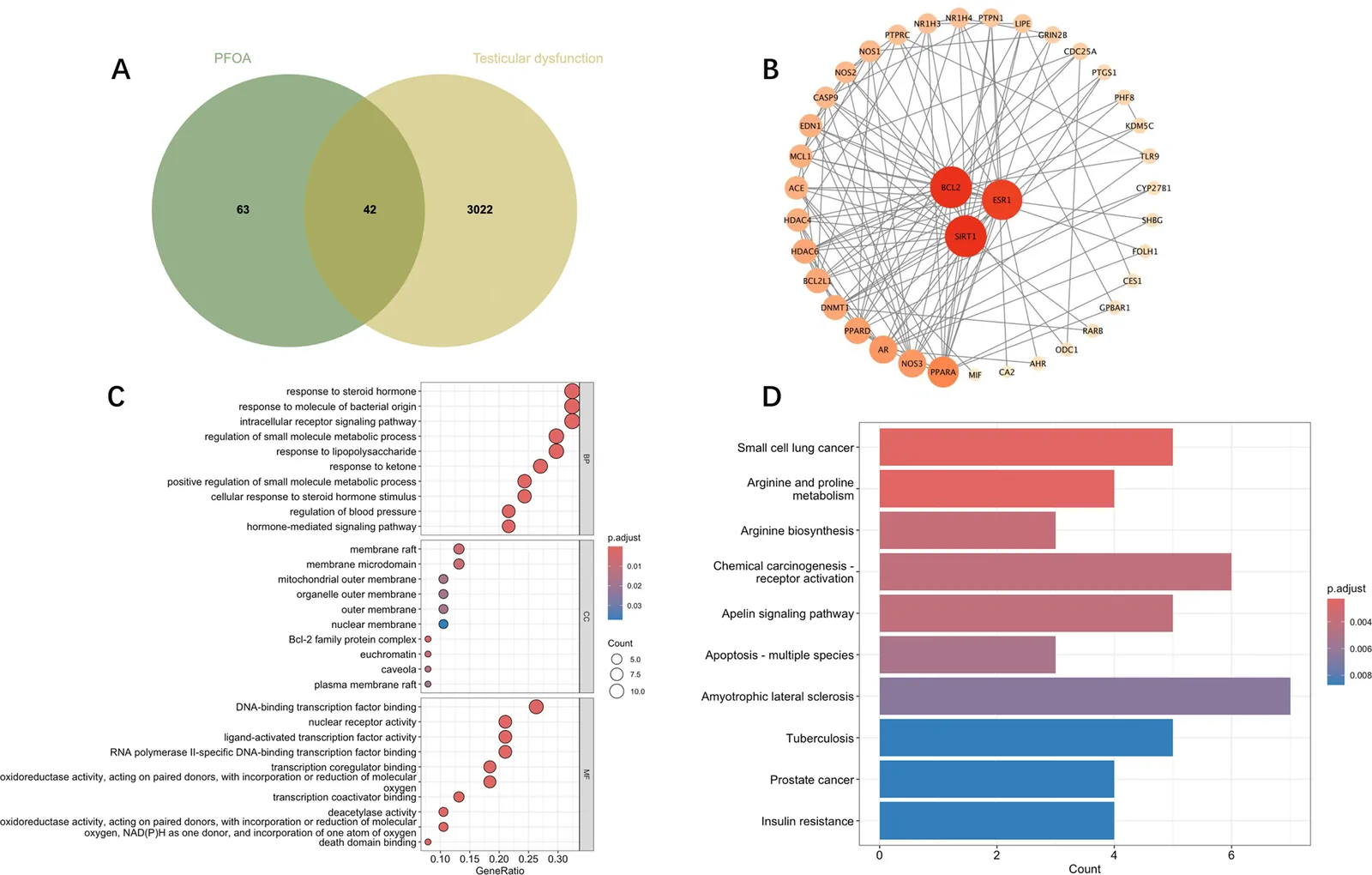
Identification and functional analysis of PFOA associated genes related to testicular dysfunction. **A.** The venn diagram illustrates the intersection of target genes associated with PFOA and testicular dysfunction related genes. **B.** PPI network of the hub genes in PFOA- testicular dysfunction related genes. **C.** GO enrichment analysis of PFOA- testicular dysfunction related genes. **D.** KEGG pathway enrichment analysis of PFOA- testicular dysfunction related genes

We obtained 105 PFOA related genes and 3,064 testicular dysfunction related genes; intersection analysis identified 42 PFOA–testicular dysfunction–related genes (Fig. 4A). PPI analysis indicated that BCL2, ESR1, and SIRT1 were key network nodes (Fig. 4B).

GO enrichment results (Fig. 4C) showed significant enrichment of PFOA associated genes in multiple biological aspects related to testicular dysfunction. In biological processes, these genes were involved in responses to steroid hormones, hormone-mediated signaling pathways, and intracellular receptor signaling, and were also enriched in immune responses to LPS and bacterial-derived molecules, responses to ketone bodies, and regulation/positive regulation of small-molecule metabolic processes. These findings suggest that PFOA may contribute to reproductive impairment through disruption of steroid hormone signaling, induction of inflammatory responses, and perturbation of testicular metabolic homeostasis. In cellular components, enriched terms included membrane rafts, membrane microdomains, nuclear membrane and outer nuclear membrane, organelle outer membrane, mitochondrial outer membrane, caveolae-rich membrane domains, and euchromatin regions, suggesting potential effects on membrane signaling platforms, mitochondrial function, and nuclear chromatin status, thereby affecting testicular cell structure and energy metabolism. In molecular functions, genes were enriched in nuclear receptor activity, ligand-activated transcription factor activity, DNA-binding transcription factor binding, RNA polymerase II–specific transcription factor binding, oxidoreductase activity, and transcription coactivator binding, indicating involvement in gene expression regulation and redox homeostasis.

KEGG enrichment analysis (Fig. 4D) revealed significant enrichment in pathways including arginine and proline metabolism, arginine biosynthesis, apelin signaling, apoptosis (multiple species), and insulin resistance. In addition, cancer related pathways (e.g., small cell lung cancer and prostate cancer) and chemical carcinogenesis–receptor activation pathways were enriched, suggesting that PFOA may disrupt testicular function through mechanisms involving altered cell proliferation, apoptosis, and metabolic homeostasis. Overall, these results indicate that PFOA exposure may damage testicular tissue via multiple mechanisms, including metabolic dysregulation, apoptosis activation, and signaling pathway perturbation.

### Potential effects of PFOA on male reproductive health

**Fig. 5:**
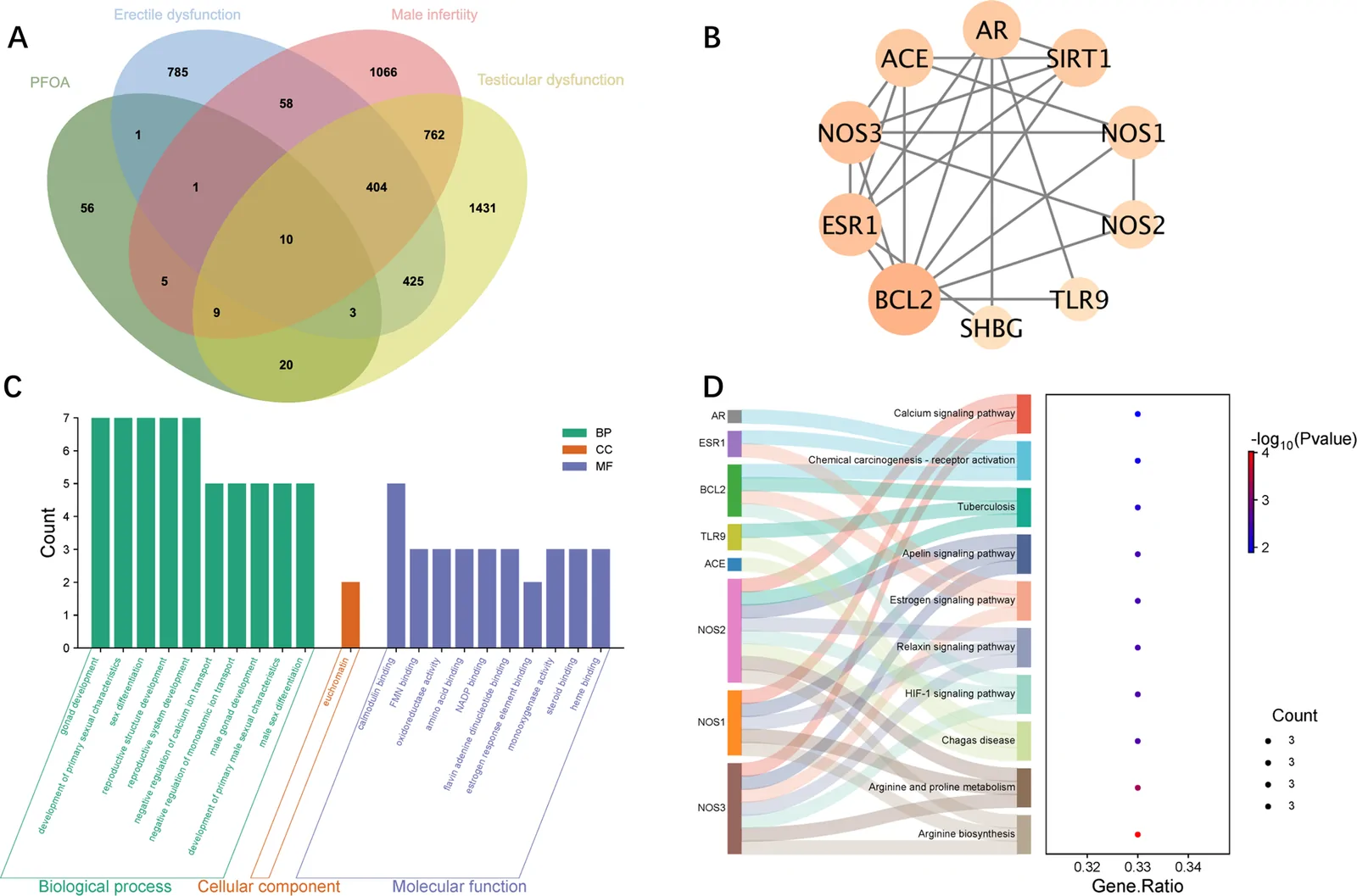
Integrated analysis of shared key genes among erectile dysfunction, male infertility, and testicular dysfunction associated with PFOA exposure. **A.** The venn diagram illustrates the intersection of target genes associated with PFOA and male reproductive health related genes. **B.** PPI network of the hub genes in PFOA- male reproductive health related genes. **C.** GO enrichment analysis of PFOA- male reproductive health related genes. **D.** KEGG pathway enrichment analysis of PFOA- male reproductive health related genes

Intersection analysis between PFOA related genes and all phenotype related gene sets identified 10 PFOA–male reproductive health related genes (Fig. 5A). The PPI results again indicated that BCL2, AR, the NOS family (NOS1, NOS2, and NOS3), and ESR1 were central network nodes, suggesting that they may play key regulatory roles in PFOA-induced reproductive impairment (Fig. 5B).

GO enrichment analysis (Fig. 5C) showed significant enrichment across biological processes, cellular components, and molecular functions. In biological processes, genes were enriched in gonadal development, development of primary male sexual characteristics, male sex differentiation, reproductive system development, and negative regulation of calcium ion transport. These findings suggest that PFOA exposure may interfere with key processes such as gonadal formation, sex differentiation, and ion homeostasis, thereby disrupting normal development and functional maintenance of the male reproductive system. In cellular components, genes were significantly enriched in the mitochondrion, implying that mitochondria may be important targets of PFOA-induced testicular cell injury and supporting roles for metabolic dysregulation and mitochondrial dysfunction in PFOA toxicity. In molecular functions, enriched terms included calcium ion binding, nuclear receptor activity, oxidoreductase activity, steroid binding, estrogen receptor binding, and nicotinamide adenine dinucleotide phosphate binding, indicating involvement in hormone signal transduction, oxidative stress regulation, and metal ion binding, which may collectively mediate PFOA-induced disturbances in testicular endocrine function, redox balance, and cellular homeostasis.

KEGG enrichment analysis (Fig. 5D) further revealed that the key genes were enriched in the calcium signaling pathway, apelin signaling pathway, estrogen signaling pathway, hypoxia-inducible factor 1 signaling pathway, relaxin signaling pathway, and arginine and proline metabolism. In addition, arginine biosynthesis and chemical carcinogenesis–receptor activation pathways were enriched. In the gene–pathway association analysis (Sankey diagram), NOS1, NOS2, NOS3, ACE, AR, ESR1, and BCL2 were connected to multiple pathways, highlighting their central roles. The NOS family was closely related to calcium signaling, hypoxia-inducible factor 1 signaling, and amino acid metabolism, suggesting that PFOA may impair testicular function by affecting NO production and cellular oxygen metabolism. Enrichment of AR and ESR1 underscores the central role of androgen and estrogen receptor signaling in male reproductive toxicity, whereas enrichment of BCL2 suggests involvement of apoptosis in PFOA-induced testicular cell injury.

### Molecular docking

**Fig. 6:**
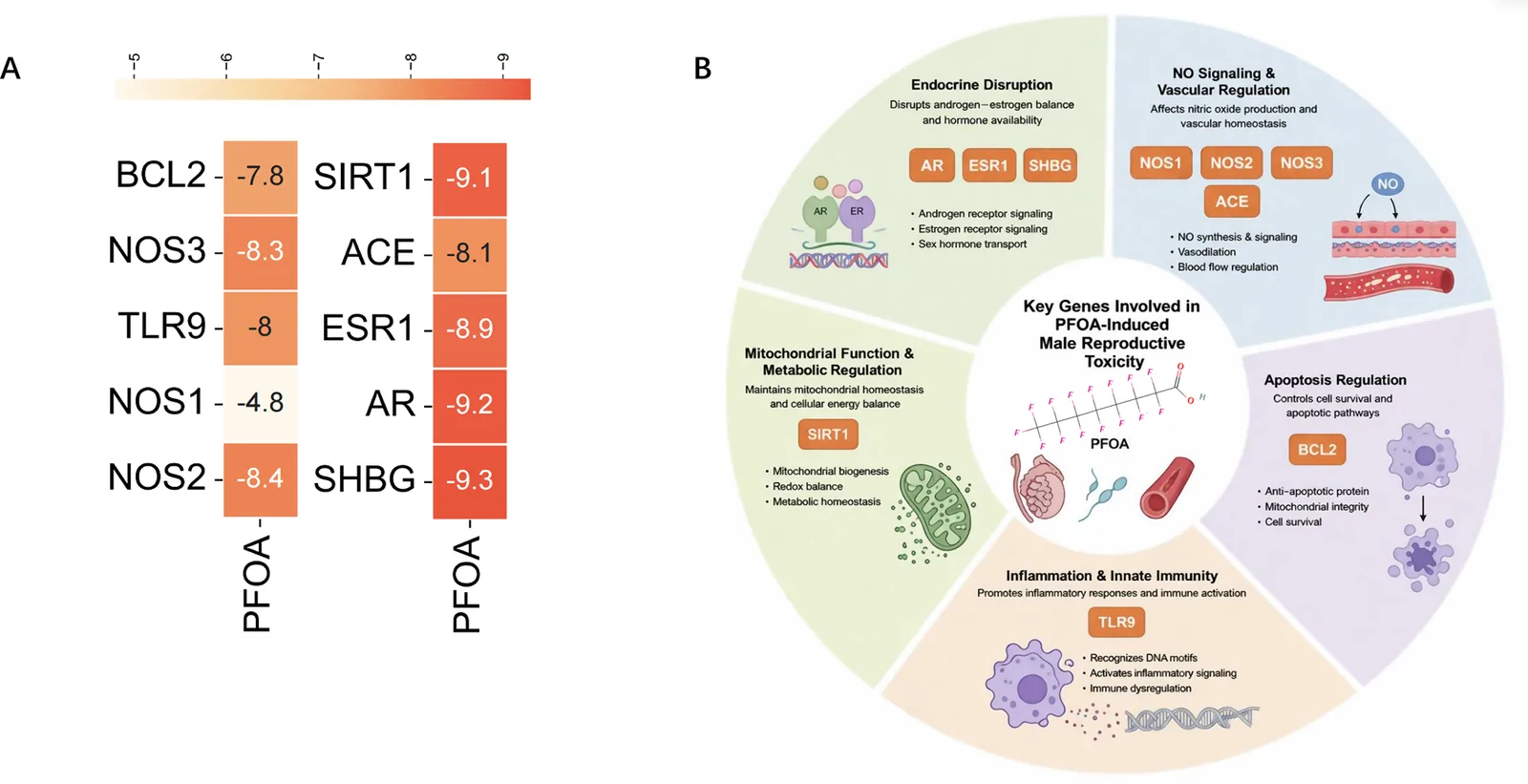
Molecular docking and integrated functional characterization of key genes involved in PFOA-induced male reproductive toxicity. **A.** Heatmap of docking binding energies between PFOA and 10 male reproductive health related genes. **B.** Integrated mechanistic schematic of the 10 shared key genes involved in PFOA-induced male reproductive toxicity

To validate direct interactions between PFOA and hub proteins, molecular docking was performed for the 10 core proteins. The results showed that, except for NOS1, the remaining proteins exhibited a strong binding tendency (Vina score < −7 kcal/mol), suggesting favorable binding stability (Fig. 6A).

Specifically, SHBG (−9.3 kcal/mol), AR (−9.2 kcal/mol), SIRT1 (−9.1 kcal/mol), and ESR1 (−8.9 kcal/mol) showed the most favorable binding energies, suggesting that PFOA may bind stably to sex hormone–binding globulin, androgen receptor, estrogen receptor, and SIRT1—key proteins involved in hormonal regulation and metabolism—supporting the possibility that PFOA may act as a potential endocrine disruptor. In addition, nitric oxide synthase family members (NOS2: −8.4; NOS3: −8.3; NOS1: −4.8 kcal/mol) showed varying degrees of binding toward PFOA. The more favorable binding of NOS2 and NOS3 suggests that PFOA may influence NO production and vascular endothelial function, consistent with its potential roles in modulating erectile function and testicular microcirculation. BCL2 (−7.8 kcal/mol), ACE (−8.1 kcal/mol), and TLR9 (−8.0 kcal/mol) also exhibited favorable binding, implying that PFOA may interfere with apoptosis regulation, the renin–angiotensin system, and innate immune pathways, thereby affecting male reproductive homeostasis and inflammatory responses.

### Functional analysis

To further integrate the potential roles of the 10 shared key genes in PFOA related male reproductive injury, we constructed a mechanistic schematic based on network toxicology, molecular docking results, and functional characteristics (Fig. 6B). The results showed that these 10 key genes could be broadly classified into five functional modules: endocrine disruption, NO signaling and vascular regulation, mitochondrial function and metabolic regulation, apoptosis regulation, and inflammatory/innate immune responses. Specifically, AR, ESR1, and SHBG were primarily involved in the regulation of androgen–estrogen balance and hormone bioavailability, suggesting that PFOA may induce endocrine dysfunction by interfering with sex hormone receptor signaling and hormone transport processes. NOS1, NOS2, NOS3, and ACE were mainly enriched in biological processes related to NO production and vascular homeostasis, indicating their potential involvement in PFOA-induced endothelial dysfunction and microcirculatory disturbance. SIRT1 was mainly associated with mitochondrial homeostasis, redox balance, and metabolic regulation. BCL2 was primarily involved in the regulation of apoptosis, whereas TLR9 was closely associated with inflammatory responses and activation of innate immunity.

### RT-qPCR

**Fig. 7:**
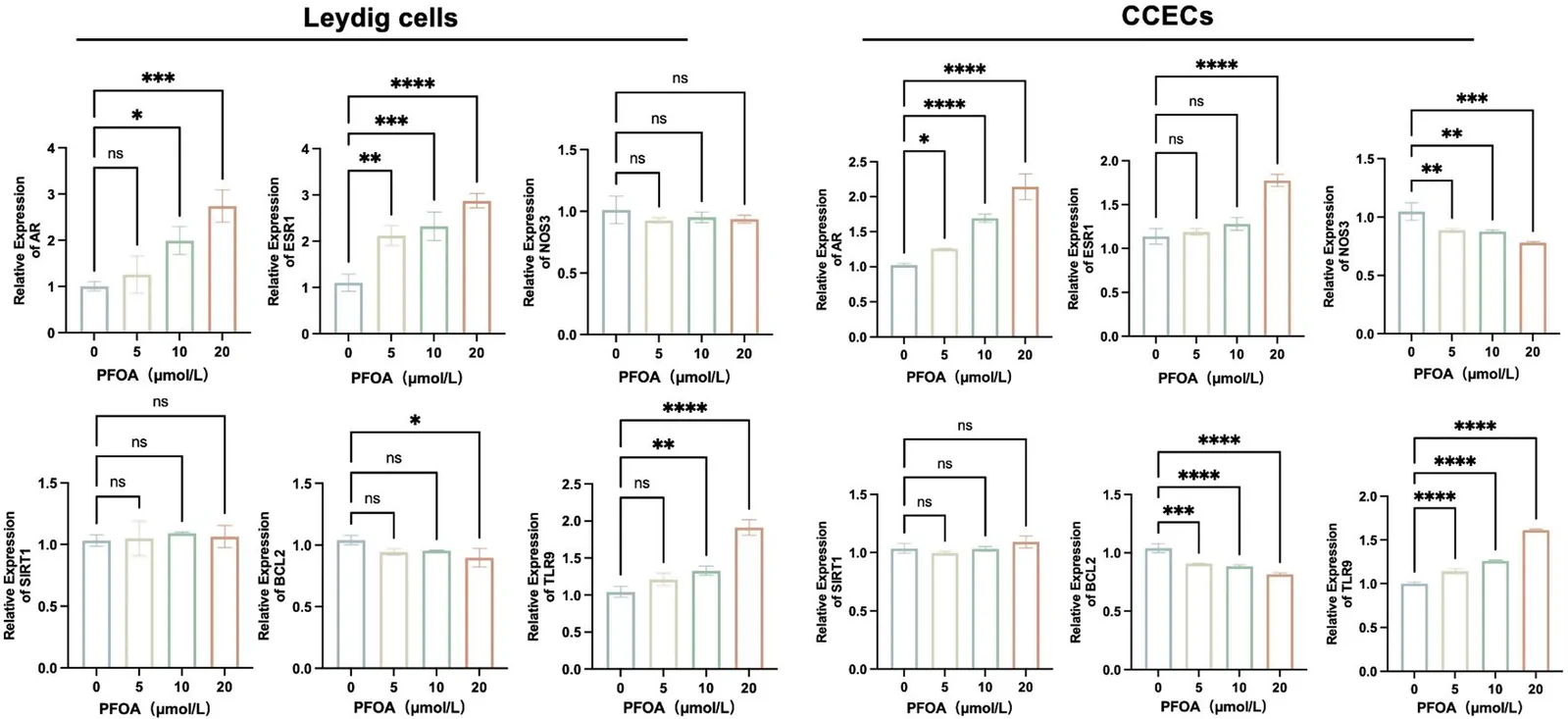
Effects of PFOA on the mRNA expression of key genes in Leydig cells and CCECs. RT-qPCR analysis of AR, ESR1, NOS3, SIRT1, BCL2, and TLR9 mRNA expression in Leydig cells and CCECs following exposure to different concentrations of PFOA (0, 5, 10, and 20 μmol/L). In Leydig cells, PFOA treatment increased the mRNA expression of AR, ESR1, and TLR9, whereas NOS3 and SIRT1 showed no significant changes. BCL2 expression exhibited a decreasing trend after PFOA exposure. In CCECs, PFOA treatment upregulated AR, ESR1, and TLR9, while NOS3 and BCL2 were downregulated, with no significant change observed in SIRT1

To validate the key targets identified through network toxicology, we further examined the mRNA expression levels of AR, ESR1, NOS3, SIRT1, BCL2, and TLR9 in Leydig cells and CCECs following treatment with different concentrations of PFOA (Fig. 7).

In Leydig cells, the mRNA expression levels of AR and ESR1 showed an overall upward trend with increasing PFOA concentrations, with the most pronounced increases observed in the high-concentration group. NOS3 expression exhibited no obvious overall change, and SIRT1 remained relatively unchanged across groups. BCL2 expression showed little variation under low- and medium-dose treatment but displayed a downward trend after high-concentration PFOA exposure. In contrast, TLR9 mRNA expression was significantly upregulated in a concentration dependent manner.

In CCECs, AR and ESR1 mRNA levels were also elevated after PFOA treatment, with the most significant increases observed in the high concentration group. Unlike that in Leydig cells, NOS3 mRNA expression in CCECs gradually decreased with increasing PFOA concentration, suggesting that PFOA may suppress endothelial NO synthesis related pathways. SIRT1 expression did not differ significantly among treatment groups. BCL2 expression progressively decreased as PFOA concentration increased, whereas TLR9 showed marked upregulation.

## Discussion

This study systematically investigated the potential molecular links between PFOA exposure and male reproductive dysfunction through an integrated strategy combining network toxicology, molecular docking, and in vitro validation. Unlike most previous studies that focused on a single endpoint, the present work incorporated three phenotypic categories—ED, male infertility, and testicular dysfunction—into a unified analytical framework, and further identified 10 shared key genes, namely AR, ESR1, BCL2, NOS1, NOS2, NOS3, ACE, SIRT1, TLR9, and SHBG. Taken together, the results from GO/KEGG enrichment, PPI network construction, molecular docking, and preliminary experiments in Leydig cells and CCECs suggest that PFOA-induced male reproductive injury is unlikely to depend on a single molecular event, but is more likely mediated by the coordinated involvement of multiple pathways, including endocrine imbalance, dysregulated NO signaling, and apoptosis–inflammation disequilibrium.

First, endocrine disruption may represent a central initiating event in PFOA related male reproductive toxicity. Accumulating evidence from both review articles and experimental studies suggests that PFOA/PFOS can disrupt male reproductive endocrine homeostasis through adverse effects on Leydig cell function, steroidogenic processes, and circulating sex hormone levels [15, 16, 28]. In the present study, AR and ESR1 occupied shared core positions across all three phenotypes and also exhibited strong binding tendencies in molecular docking analyses. Meanwhile, SHBG showed one of the most favorable binding energies among all core proteins, suggesting that PFOA may not only act on hormone receptors themselves, but may also influence the circulating bioavailability of sex hormones and their receptor exposure levels [29–31]. Network enrichment analysis further revealed that the core genes were significantly associated with nuclear receptor activity, ligand-activated transcription factor activity, steroid binding, hormone-mediated signaling pathways, and the estrogen signaling pathway. These findings indicate that PFOA may disrupt androgen–estrogen balance and downstream transcriptional regulatory networks, thereby affecting gonadal development, male sexual differentiation, and the maintenance of reproductive system function [32]. More importantly, in the in vitro experiments, both AR and ESR1 showed increased mRNA expression after PFOA exposure in both Leydig cells and CCECs, which experimentally supports the possibility that PFOA induces endocrine related abnormal activation. It should be emphasized that such upregulation does not necessarily indicate physiological enhancement, but may instead reflect stress associated remodeling or imbalanced activation of receptor networks, ultimately leading to disruption of hormonal homeostasis.

Second, abnormal NO signaling and impairment of vascular/microcirculatory homeostasis may serve as an important bridge linking ED and testicular dysfunction. Accumulating evidence indicates that endothelial dysfunction is a central event in the pathogenesis of ED, whereas reduced NOS3 expression or activity and diminished NO bioavailability are considered key pathological determinants of impaired penile vascular relaxation [33–35]. In the present study, the NOS family occupied a prominent position in the shared core network and was closely associated with arginine biosynthesis, arginine and proline metabolism, calcium signaling, the HIF-1 signaling pathway, the relaxin signaling pathway, and the apelin signaling pathway. Previous studies have shown that arginine metabolism provides a key substrate source for NO synthesis, calcium signaling participates in the regulation of NOS activity, and HIF-1, relaxin, and apelin pathways are all closely related to endothelial homeostasis, blood flow regulation, and vasodilation. Therefore, at the network level, it is reasonable to speculate that PFOA may impair endothelial function and erection related hemodynamics by interfering with NO production and its upstream regulatory processes at multiple levels. In the molecular docking analysis, both NOS2 and NOS3 displayed relatively stable binding tendencies, further supporting the possibility that PFOA may directly or indirectly affect the NO axis. Notably, in the in vitro validation, NOS3 showed a downward trend in CCECs after PFOA exposure, whereas no obvious change was observed in Leydig cells. This difference suggests that the effects of PFOA on NO related injury may exhibit clear cell-type specificity: in CCECs, which primarily reflect endothelial function, PFOA is more likely to manifest as NOS3 inhibition and impaired NO signaling, whereas in Leydig cells, the dominant changes may be more related to endocrine and inflammatory disturbances. This observation is consistent with the pathological features of ED, which rely heavily on endothelial dysfunction and impaired vasodilation, and further strengthens the biological plausibility that PFOA may contribute to ED development.

Third, dysregulation of apoptosis and inflammation may represent a common terminal effector pathway in PFOA-induced male reproductive injury. In the present study, repeated enrichment of apoptosis- and inflammation/immune related signaling was observed across multiple phenotypes, among which BCL2 and TLR9 deserve particular attention. BCL2 is a classical anti-apoptotic molecule, showing a high degree of connectivity in the PPI network, and exhibiting favorable binding stability in molecular docking analysis, suggesting that it may be an important node through which PFOA regulates the cellular survival threshold. On the other hand, TLR9, an innate immune recognition related molecule, also occupied a key position in the shared network. Previous studies have generally suggested that TLR9 is involved in inflammatory sensing and activation of downstream pathways such as NF-κB, and that its abnormal activation may promote chronic inflammation or immune disequilibrium in local tissues [36, 37]. As illustrated in the mechanistic integration model in Fig. 6, PFOA may simultaneously weaken anti-apoptotic capacity and enhance inflammatory responses in reproductive related cells, thereby accelerating disruption of cellular homeostasis and amplifying injury.

In addition, SIRT1-mediated mitochondrial and metabolic dysregulation may provide an important background mechanism underlying the above injury processes. Network analysis showed that SIRT1 was one of the shared core genes and was closely related to mitochondrial localization, redox processes, and metabolic homeostasis. According to the integrated mechanistic framework shown in Fig. 6, SIRT1 was mainly assigned to the module of “mitochondrial function and metabolic regulation,” suggesting that it may participate in PFOA-induced disturbances in energy metabolism and oxidative stress. Although no significant changes in SIRT1 expression were observed in either cell type in the present in vitro experiments, this does not necessarily indicate that SIRT1 is uninvolved in the injury process. On the one hand, SIRT1 may be more accurately reflected by alterations in functional activity, subcellular localization, or downstream regulatory effects, rather than by large fluctuations in expression level alone. On the other hand, the current treatment duration and concentration settings may have been more suitable for revealing the direct effects of PFOA on AR, ESR1, NOS3, BCL2, and TLR9, while being insufficient to capture the dynamic changes in SIRT1. Therefore, SIRT1 may function more as a background regulator rather than an early-response molecule in PFOA related male reproductive toxicity, and its specific role still requires further investigation in combination with ROS, mitochondrial membrane potential, and energy metabolism related indicators.

Taken together, the above evidence led us to propose a multi-pathway synergistic model in which PFOA may impair male reproductive health through “hormonal imbalance–aberrant NO signaling–dysregulated apoptosis and inflammation.” Specifically, PFOA may first act on the AR/ESR1/SHBG related endocrine axis, disrupting the balance between estrogen and androgen signaling as well as receptor-mediated transcriptional regulation. It may subsequently affect NO production and vascular/microcirculatory function through NOS family members, calcium signaling, and arginine metabolism. In the context of metabolic stress and mitochondrial dysfunction, PFOA may further induce inflammatory responses and disturb apoptotic regulation, ultimately leading to multiple phenotypic outcomes across different tissues, including erectile dysfunction, male infertility, and testicular functional impairment.

It should be noted that this study still has several inherent limitations typical of network toxicology research. First, PFOA related targets were mainly obtained from database retrieval and algorithm-based prediction, and may therefore be affected by data coverage, evidence levels, and model bias. Moreover, these data lack information on dose, exposure duration, and tissue specificity. Second, the disease related gene sets were derived from GeneCards and filtered using a relevance score threshold. Although this strategy may improve the signal-to-noise ratio, it may also exclude genes with low scores but potentially important regulatory roles. In addition, molecular docking only provides a structural-level inference of binding tendency; therefore, molecular dynamics simulations and further experimental validation are needed to improve the reliability and reproducibility of the docking results. Finally, future animal experiments and large-scale epidemiological studies are required to validate our conclusions.

## Conclusions

This study identifies potential key targets and pathways underlying PFOA associated male reproductive health impairment at a systems biology level, highlighting the central role of endocrine-disrupting signaling across multiple reproductive endpoints. The results further suggest that dysregulation of the NO axis and activation of inflammation/apoptosis pathways may synergize with hormonal disturbances to promote the progression of male reproductive injury. Although further experimental validation is required, these findings nominate specific candidate targets and provide a framework to inform prevention strategies and public health risk assessment for male reproductive health.

## Data Availability

No datasets were generated or analysed during the current study.

## References

[1] Kenney LB, Antal Z, Ginsberg JP, Hoppe BS, Bober SL, Yu RN, et al. Improving Male reproductive health after childhood, adolescent, and young adult cancer: progress and future directions for survivorship research. J Clin Oncol. 2018;36(21):2160–68. Epub 20180606. PubMed PMID: 29874140. doi: 10.1200/jco.2017.76.3839. 29874140

[2] Skakkebaek NE, Rajpert-De Meyts E, Buck Louis GM, Toppari J, Andersson AM, Eisenberg ML, et al. Male reproductive disorders and fertility trends: influences of environment and genetic susceptibility. Physiol Rev. 2016;96(1):55–97. PubMed PMID: 26582516; PubMed Central PMCID: PMC4698396. 10.1152/physrev.00017.2015. PMC4698396 26582516

[3] Segal TR, Giudice LC. Systematic review of climate change effects on reproductive health. Fertil Steril. 2022;118(2):215–23. PMID: 35878942. 10.1016/j.fertnstert.2022.06.005.PubMed. 35878942

[4] Luche S, Fanelli A, Buschini A, Ricci A, Maccari C, Andreoli R, et al. PFOA and PFOS accumulation induces genotoxic damage and proteomic alterations in cannabis sativa shoots. J Hazard Mater. 2025;501:140686. Epub 20251201. PMID: 41344189. doi: 10.1016/j.jhazmat.2025.140686.PubMed. 41344189

[5] van Loon S, Xie G, Svendsen C, Kraak MHS, de Jeu L, Schut NC, et al. Microplastics and PFAS as ubiquitous pollutants affect potencies of highly toxic chemicals in mixtures. J Hazard Mater. 2025;500:140493. Epub 20251121. PMID: 41313859. doi: 10.1016/j.jhazmat.2025.140493.PubMed. 41313859

[6] Stavros S, Kathopoulis N, Moustakli E, Potiris A, Anagnostaki I, Topis S, et al. Endocrine-disrupting chemicals and male infertility: mechanisms, risks, and regulatory challenges. J Xenobiot. 2025;15(5). Epub 20251013. doi: 10.3390/jox15050165.PubMed PMID: 41149751; PubMed Central PMCID: PMC12565704.165. 10.3390/jox15050165. PMC12565704 41149751

[7] Liu Z, Li Y, Wang J, Zeng Y, Wu Q, Zhu X, et al. Network toxicology and machine learning reveal the impact of acetyl tributyl citrate on erectile dysfunction and identify cathepsin S as a critical regulator. Reprod Toxicol. 2025;139:109101. Epub 20251102. PubMed PMID: 41187890. doi: 10.1016/j.reprotox.2025.109101. 41187890

[8] Zhang S, Qin D, Lin Z, Wang S, Wang L, Wang J, et al. BPP disrupts spermatogenesis via mitochondrial-ER stress-mediated autophagic flux blockade in mice. Ecotoxicol Environ Saf. 2025;307:119392. Epub 20251114.PubMed PMID: 41240577. doi: 10.1016/j.ecoenv.2025.119392. 41240577

[9] Jiang P, Wang W, Li J, Li Y, Ji X, Yue H. The effects of bisphenol compounds on endocrine disruption and reproductive function from epidemiological analysis to animal exposure: a mixture analysis. J Environ Sci (China). 2026;160:537–47. Epub 20250417. PMID: 41177635. doi:10.1016/j.jes.2025.04.042. 41177635

[10] Kumaresan A, Yadav P, Hofmann A, Sinha MK, Ebenezer Samuel King JP, Nag P, et al. Unveiling the reproductive risks of PFOS: testicular proteome disruption and hormonal imbalance in male rats†. Biol Reprod. 2025;113(3):686–703. PMID: 40709786. 10.1093/biolre/ioaf167. PMC13369134 40709786

[11] Liu D, Yan S, Wang P, Chen Q, Liu Y, Cui J, et al. Perfluorooctanoic acid (PFOA) exposure in relation to the kidneys: a review of current available literature. Front Physiol. 2023;14:1103141. Epub 20230126. PMID: 36776978; PubMed Central PMCID: PMC9909492. doi: 10.3389/fphys.2023.1103141. PMC9909492 36776978

[12] Post GB, Cohn PD, Cooper KR. Perfluorooctanoic acid (PFOA), an emerging drinking water contaminant: a critical review of recent literature. Environ Res. 2012;116:93–117. Epub 20120504. PMID: 22560884. doi: 10.1016/j.envres.2012.03.007. 22560884

[13] Ilieva Z, Hania P, Suehring R, Gilbride K, Hamza R. Impact of perfluorooctanoic acid (PFOA) and perfluorooctane sulfonic acid (PFOS) on secondary sludge microorganisms: removal, potential toxicity, and their implications on existing wastewater treatment regulations in Canada. Environ Sci Process Impacts. 2023;25(10):1604–14. Epub 20231018. PMID: 37674406. doi: 10.1039/d3em00202k. 37674406

[14] Wang Y, Aimuzi R, Nian M, Zhang Y, Luo K, Zhang J. Perfluoroalkyl substances and sex hormones in postmenopausal women: NHANES 2013-2016. Environ Int. 2021;149:106408. Epub 20210203. PMID: 33548847. doi: 10.1016/j.envint.2021.106408. 33548847

[15] Shi W, Zhang Z, Li M, Dong H, Li J. Reproductive toxicity of PFOA, PFOS and their substitutes: a review based on epidemiological and toxicological evidence. Environ Res. 2024;250:118485. Epub 20240217. PubMedPMID: 38373549. doi: 10.1016/j.envres.2024.118485. 38373549

[16] Wang Z, Zhang T, Wu J, Wei X, Xu A, Wang S, et al. Male reproductive toxicity of perfluorooctanoate (PFOA): rodent studies. Chemosphere. 2021;270:128608. Epub 20201014. PubMedPMID: 33081999. doi: 10.1016/j.chemosphere.2020.128608. 33081999

[17] Calvert L, Green MP, De Iuliis GN, Dun MD, Turner BD, Clarke BO, et al. Assessment of the emerging threat posed by Perfluoroalkyl and polyfluoroalkyl substances to Male reproduction in humans. Front Endocrinol (Lausanne). 2021;12:799043. Epub 20220309. PMID: 35356147; PubMed Central PMCID: PMC8959433. doi: 10.3389/fendo.2021.799043. PMC8959433 35356147

[18] Pavani R, Venkaiah K, Prakasam PG, Dirisala VR, Krishna PG, Kishori B, et al. Protective effects of resveratrol against perfluorooctanoic acid-induced testicular and epididymal toxicity in adult rats exposed during their prepubertal period. Toxics. 2025;13(2). Epub 20250129. doi: 10.3390/toxics13020111.PubMed PMID: 39997926; PubMed Central PMCID: PMC11860439.111. 10.3390/toxics13020111. PMC11860439 39997926

[19] Yang H, Lou C, Sun L, Li J, Cai Y, Wang Z, et al. admetSAR 2.0: web-service for prediction and optimization of chemical ADMET properties. Bioinformatics. 2019;35(6):1067–69. PMID: 30165565. 10.1093/bioinformatics/bty707. 30165565

[20] Yu T, Zhang H, Xie J, Yu Y, Zhang D, Wu Y, et al. Deciphering the toxicological mechanisms of per- and polyfluoroalkyl substances (PFAS) in musculoskeletal disorders via integrated network toxicology and molecular docking. Environ Int. 2025;206:109934. Epub 20251119. PMID: 41270661. doi: 10.1016/j.envint.2025.109934. 41270661

[21] Wang Y, Li C. A network toxicology approach to elucidate shared oncogenic pathways of Aristolochic acids in prostate, kidney, and bladder cancers. Toxicol Res (Camb). 2025;14(5):tfaf150. Epub 20251030. PMID: 41181379; PubMed Central PMCID: PMC12572709. doi: 10.1093/toxres/tfaf150. PMC12572709 41181379

[22] Li Y, Liu Z, Zhou T, Zhu X, Wu Q, Zeng Y, et al. Integrating network toxicology and Mendelian randomization to uncover the role of AHR in linking air pollution to male reproductive health. Reprod Toxicol. 2025;135:108918. Epub 20250414. PMID: 40239776. doi: 10.1016/j.reprotox.2025.108918. 40239776

[23] Li Y, Wang J, Liu Z, Zhu X, Wu Q, Meng C, et al. Identification of key genes involved in phthalate-induced male erectile dysfunction: insights from network toxicology and bioinformatics analyses. Reprod Toxicol. 2025;137:109026. Epub 20250806. PMID: 40780689. doi: 10.1016/j.reprotox.2025.109026. 40780689

[24] Meng C, Li Y, Zhou T, Zhu X, Li Y, Tan X, et al. Artificial sweeteners and male infertility: network toxicology and experimental evidence reveal FGFR1 as the critical target. Reprod Toxicol. 2026;141:109191. Epub 20260217. PMID: 41713522. doi: 10.1016/j.reprotox.2026.109191. 41713522

[25] Tang H, Finn RD, Thomas PD. TreeGrafter: phylogenetic tree-based annotation of proteins with gene ontology terms and other annotations. Bioinformatics. 2019;35(3):518–20. PMID: 30032202; PubMed Central PMCID: PMC6361231 10.1093/bioinformatics/bty625. PMC6361231 30032202

[26] Kanehisa M, Goto S. KEGG: kyoto encyclopedia of genes and genomes. Nucleic Acids Res. 2000;28(1):27–30. PubMed PMID: 10592173; PubMed Central PMCID: PMC102409. 10.1093/nar/28.1.27. PMC102409 10592173

[27] Li Y, Zhou T, Liu Z, Zhu X, Wu Q, Meng C, et al. Air pollution and prostate cancer: unraveling the connection through network toxicology and machine learning. Ecotoxicol Environ Saf. 2025;292:117966. Epub 20250228. PubMed PMID: 40022828. doi: 10.1016/j.ecoenv.2025.117966. 40022828

[28] Tarapore P, Ouyang B. Perfluoroalkyl chemicals and male reproductive health: do PFOA and PFOS increase risk for male infertility? Int J Environ Res Public Health. 2021;18(7). Epub 20210405. doi: 10.3390/ijerph18073794.PubMed PMID: 33916482; PubMed Central PMCID: PMC8038605.3794. 10.3390/ijerph18073794. PMC8038605 33916482

[29] Fang YW, Chen CW, Lin HC, Su TC, Wang C, Lin CY. Associations between PFAS exposure and HPG axis hormones in U.S. Women. Life (Basel). 2025;15(12). Epub 20251216. doi: 10.3390/life15121923.PubMed PMID: 41465861; PubMed Central PMCID: PMC12734433. 10.3390/life15121923. PMC12734433 41465861

[30] Lagostena L, Magnelli V, Rotondo D, Dondero F. Persistent pollutants and the developing brain: the role of PFAS in neurodevelopmental disorders. Front Cell Neurosci. 2025;19:1696173. Epub 20251121. PubMed PMID: 41356495; PubMed Central PMCID: PMC12678283. doi: 10.3389/fncel.2025.1696173. PMC12678283 41356495

[31] van Larebeke N, Cox B, Remy S, Voorspoels S, Den Hond E, Colles A, et al. Per- and polyfluoroalkyl substances (PFAS), thyroid hormones, sexual hormones and pubertal development in adolescents residing in the neighborhood of a 3M factory. Environ Health. 2025;24(1):34. Epub 20250607. PubMed PMID: 40483423; PubMed Central PMCID: PMC12144784. doi: 10.1186/s12940-025-01188-1. PMC12144784 40483423

[32] Zhang D, Hu J, Li H. Perfluorooctanoic acid inhibits androgen biosynthesis in rat immature leydig cells. Environ Toxicol. 2024;39(3):1700–14. Epub 20231205. PubMed PMID: 38050817. doi: 10.1002/tox.24042. 38050817

[33] Bivalacqua TJ, Liu T, Musicki B, Champion HC, Burnett AL. Endothelial nitric oxide synthase keeps erection regulatory function balance in the penis. Eur Urol. 2007;51(6):1732–40. Epub 20061110. PubMed PMID: 17113219; PubMed Central PMCID: PMC3328507. doi: 10.1016/j.eururo.2006.10.061. PMC3328507 17113219

[34] Toda N. Age related changes in endothelial function and blood flow regulation. Pharmacol Ther. 2012;133(2):159–76. Epub 20111029. PubMed PMID: 22079549. doi: 10.1016/j.pharmthera.2011.10.004. 22079549

[35] Kaltsas A, Zikopoulos A, Dimitriadis F, Sheshi D, Politis M, Moustakli E, et al. Oxidative stress and erectile dysfunction: pathophysiology, impacts, and potential treatments. Curr Issues Mol Biol. 2024;46(8):8807–34. Epub 20240814. PubMed PMID: 39194738; PubMed Central PMCID: PMC11353036. 10.3390/cimb46080521. 10.3390/cimb46080521 PMC11353036 39194738

[36] Saber MM, Monir N, Awad AS, Elsherbiny ME, Zaki HF. TLR9: a friend or a foe. Life Sci. 2022;307:120874. Epub 20220810. PubMed PMID: 35963302. doi: 10.1016/j.lfs.2022.120874. 35963302

[37] Rogers GL, Martino AT, Aslanidi GV, Jayandharan GR, Srivastava A, Herzog RW. Innate immune responses to AAV vectors. Front Microbiol. 2011;2:194. Epub 20110919. PubMed PMID: 21954398; PubMed Central PMCID: PMC3175613. doi: 10.3389/fmicb.2011.00194. PMC3175613 21954398

